# Probing crustal anisotropy by receiver functions at the deep continental drilling site KTB in Southern Germany

**DOI:** 10.1111/1365-2478.12883

**Published:** 2019-10-14

**Authors:** Irene Bianchi, Götz Bokelmann

**Affiliations:** ^1^ Department of Meteorology and Geophysics University of Vienna Wien Austria; ^2^ Istituto Nazionale di Geofisica e Vulcanologia (INGV) Sezione Roma1 Rome Italy

**Keywords:** Anisotropy, Passive method, Shear wave velocity

## Abstract

Seismic anisotropy is a unique observational tool for remotely studying deformation and stress within the Earth. Effects of anisotropy can be seen in seismic data; they are due to mineral alignment, fractures or layering. Seismic anisotropy is linked to local stress and strain, allowing modern geophysics to derive geomechanical properties from seismic data for supporting well planning and fracking. For unravelling anisotropic properties of the crust, the teleseismic receiver functions methodology has started to be widely applied recently due to its ability in retrieving the three‐dimensional characteristics of the media sampled by the waves. The applicability of this technique is tested here by a field test carried out around the Kontinental Tiefbohrung site in southeastern Germany. We compare our results to previous investigations of the metamorphic rock pile of the Zone Erbendorf‐Vohenstrauss, drilled down to 9 km depth, which sampled an alternating sequence of paragneiss and amphibolite, in which a strong foliation has been produced by ductile deformation. The application of the receiver functions reveals the presence of two distinct anisotropic layers within the metamorphic rock pile at 0–4 km and below 6 km depth, with up to 8% anisotropy; the depth of these two layers corresponds to the location of mica‐rich paragneiss which show intense foliation, and finally proves the relation between the signal in the receiver functions, rock texture and presence of cracks. We have now the capability of providing insights from passive seismic data on geomechanical properties of the rocks, useful for geological exploration and engineering purposes, which will help influencing expensive drilling decisions thanks to future application of this seismic technique.

## 1 INTRODUCTION

**Figure 1:**
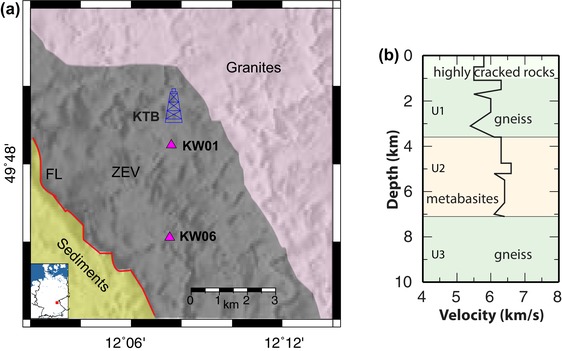
(a) Map of the KTB area showing the location of the two main stations analysed in this work with respect to the location of the KTB drilling site. (b) (R3) schematization of the lithological profile encountered during drilling, and *Vp* velocity extracted from the sonic log (both from Berckhemer *et al*. 1997).

Seismic anisotropy and its determination is of great interest because it is a key for constraining preferred alignment of structures within the Earth such as due to sedimentary layering, stress‐induced cracks and/or dykes and sills (Babuška and Cara 1991), the causes of which include the mechanical stress field and deformation. The receiver functions (RF) are time series composed of P‐to‐S phases generated at impedance contrasts at depth and their multiples. Their arrival times depend on both velocity in the crossed medium and depth of the velocity contrast (Langston 1979; Ammon 1991). The RF technique has been employed for inferring the presence of anisotropic media in the subsurface layers (e.g. Levin and Park 1998; Girardin and Farra 1998; Schulte‐Pelkum *et al*. 2005; Licciardi *et al*. 2018). To fully prove the technique of anisotropic RF, we have established a critical test – a field experiment around the deep drilling site Kontinental Tiefbohrung (KTB) in the Oberpfalz area in Bavaria (Southern Germany, Fig. 1a), to reproduce the structural information that has previously been obtained by drilling and the more classical seismic techniques, for example near‐vertical and wide‐angle seismics. The crust at the KTB site was indeed explored by means of seismic reflection studies (DEKORP Research Group 1987, 1988; Eisbacher, Lueschen and Wickert 1989; Lüschen *et al*. 1996; Harjes *et al*. 1997; DEKORP and Orogenic processes Working Groups 1999; Muller, Janik and Harjes 1999). The metamorphic body of the Zone of Erbendorf‐Vohenstrauss (ZEV) was drilled until reaching a depth of 9101 m (Harjes *et al*. 1997 and references therein). The ZEV is made of an alternating sequence of paragneisses and amphibolites showing a strong foliation.

Unexpected though was the steep inclination of that pervasive foliation, which did not correspond to previous interpretations of flat seismic reflections and mapped surface geology (Harjes *et al*. 1997). Previous tectonic interpretations had to be strongly modified, to explain the *in situ* information from the borehole (Emmermann and Lauterjung 1997; Obrien *et al*. 1997). Principal results from the drilling and accompanying geophysical experiments are described in a special JGR volume (Emmermann 1989; Haak and Jones 1997), the KTB borehole is not any longer active.

Cores samples have been used in laboratory tests for determining the seismic velocities and anisotropy (Kern and Schmidt 1990; Kern, Schmidt and Popp 1991; Zang *et al*. 1996; Berckhemer *et al*. 1997); vertical seismic profiling and multiple‐azimuth shear‐wave experiments have been performed targeting the estimation of *in situ* anisotropy (Rabbel 1994; Muller *et al*. 1999; Okaya *et al*. 2004; Rabbel *et al*. 2004). In our experiment, we recover structural information as well as anisotropy of the upper crust using the receiver function technique. This retrieved information is the basis for comparing the outcome from RF analysis in terms of amount and orientation of anisotropy, together with information of rock samples down to 9 km depth, and with high‐frequency seismic experiments around the drill (Bianchi *et al*. 2015a).

## 2 ANISOTROPY

Strong seismic anisotropy has been associated to metamorphic processes in several studies (e.g. Christensen 1965; Barruol and Mainprice 1993; Christensen and Mooney 1995; Lloyd *et al*. 2009; Almqvist and Mainprice 2017; Okaya *et al*. 2019). Also, seismic anisotropy has been observed in association to aligned cracks that occur in the vicinity of major faults or due to upper crustal stress fields (e.g. Anderson, Minster and Cole 1974; Savage *et al*. 2010; Almqvist and Mainprice 2017).

Effects of seismic anisotropy within the crust have been often observed in body and surface waves (e.g. Ozacar and Zandt 2004; Sherrington, Zandt and Frederiksen 2004; Bostock and Christensen 2012; Bianchi *et al*. 2016; Okaya *et al*. 2016). We want to draw special attention on receiver function studies, which have observed the pattern of the converted Ps waves for several anisotropic scenarios (e.g. Levin and Park 1998; Schulte‐Pelkum *et al*. 2005; Eckardt and Rabbel 2011; Piana Agostinetti *et al*. 2011; Schulte‐Pelkum and Mahan 2014; Audet 2015; Bianchi, Bokelmann and Shiomi 2015b and many others).

The location of our seismic experiment has been selected specifically for the presence of the zone of Erbendorf‐Vohenstrauss (ZEV) metamorphic body which has been drilled down to 9 km depth, and investigated both *in situ* and in laboratory on core samples. The lithological profile of the drilling (Fig. 1b) has been subdivided into three main lithological units as follows: a first unit (U1) from 0 to 3.2 km depth consisting of paragneiss containing minor intercalations of amphibolite. A second unit (U2) from 3.2 to 7.3 km depth composed of amphibolite with intercalations of metagabbros and minor intercalations of gneiss. In the third unit (U3) below 7.3 km depth, the amount of paragneiss prevails as well, reaching the bottom of the drilling hole, at 9.1 km depth (Berckhemer *et al*. 1997). Anisotropy has been detected as highest in the gneisses (U1 and U3), whereas the amphibolites and metagabbros (U2) show significant lower anisotropy (Kern *et al*. 1991); it has been shown on core samples from these units that the anisotropy reduces drastically for increasing confining pressures (Zang *et al*. 1996). Laboratory tests on gneiss samples (biotite bearing) from the ZEV have shown a marked splitting of the shear waves, where the fast wave shows polarization parallel to the foliation, whereas the slow wave is polarized normal to it (Kern *et al*. 1991).

Previous investigations in our study location have shown that the polarization of the fast shear wave is nearly NW–SE down to approximately 4 km depth (i.e. within the shallow gneiss layer), and is coinciding with the strike direction of the rock foliation (Rabbel 1994). The information of rock foliation and fractures obtained by the analysis of core samples (Röhr *et al*. 1990) of the Kontinental Tiefbohrung (KTB) pilot hole (Emmermann 1989), which reached the depth of about 4 km, were associated to the differential velocity of the fast and slow shear waves detected via vertical seismic profiling in Rabbel (1994). He noticed that the velocity of both P‐ and fast S‐waves diminishes with the decrease of the dip angle of the foliation, whereas the velocity of the slow S‐wave is constant. This is the typical behaviour of the body waves propagating within a hexagonally symmetric medium (e.g. Postma 1955). Another argument for hexagonal symmetry comes from Stroh *et al*. (1990), which detected nearly 30% of well‐oriented mica (having hexagonal elastic symmetry) in the gneiss units of the ZEV; the amount of anisotropy in the U1 has been estimated to reach 10% (Rabbel 1994).

Seismic anisotropy has been recognized to be higher at shallow depths (low pressure, i.e. up to 200 MPa) due to the positive interference of oriented microcracks and texture of the rocks. In particular, Kern *et al*. (1991) find that microfractures are parallel to the morphological sheet planes in the gneisses extracted from the pilot hole.

Moreover, encountered horizontal stress direction in the borehole are around N150° (Brudy *et al*. 1997; Plenefisch and Bonjer 1997), which is not far from the foliation strike and direction of the fast anisotropy; according to Kern, Popp and Schmidt (1994), the deviatoric stress field determined at the KTB drilling site might contribute to the seismic anisotropy *in situ*.

## 3 DATA

We selected good teleseismic events from epicentral distances (Δ) of 30°–100° and magnitude *M_b_* > 5.5 recorded at two broadband seismic stations deployed for this purpose (see the description of the experiment in Bianchi *et al*. 2015a).

**Figure 2:**
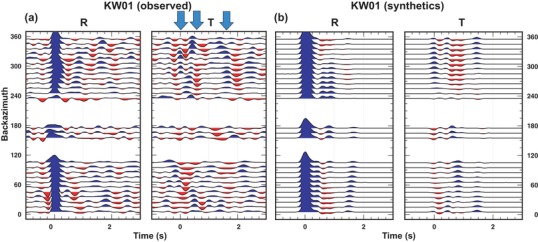
Comparison between observed (left) and synthetic (right) receiver functions calculated for station KW01. Synthetics have been computed for the model shown in Table 1. Blue arrows on the *T* component show the pulses associated with anisotropy.

**Figure 3:**
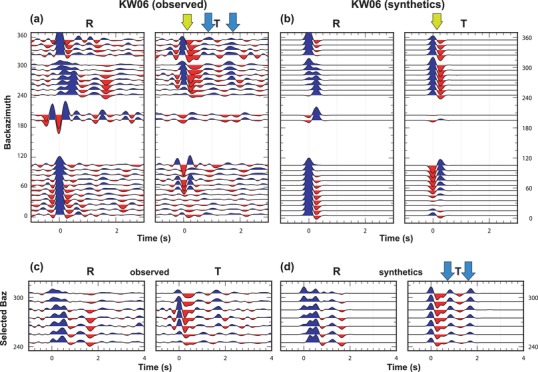
Comparison between observed (left) and synthetic (right) receiver functions calculated for station KW06. Upper panels show the whole backazimuthal sweep, whereas lower panels show the selected backazimuthal directions. Synthetics have been computed for the model shown in Table 2. Yellow arrows show the pulse associated to a shallow dipping interface, whereas blue arrows show the pulses associated with anisotropy.

The amount of collected teleseismic traces allows a reasonable backazimuthal coverage (Figs 2 and 3): events occurred between Δ of 90° and 100° have been included in order to increase the backzimuthal coverage towards the SW direction. The receiver function (RF) data sets were obtained by deconvolution of the vertical from the horizontal recordings into the radial, transverse and vertical coordinate system, where the radial (R) is computed along the great circle path between the epicentre and the station, positive away from the source, and the transverse (T) direction is calculated 90° clockwise from R. The deconvolution was performed in the frequency domain (Langston 1979; Ammon, Randall and Zandt 1990; Ammon 1991), following the approach proposed by Park and Levin (2000), applying a Slepian taper to limit the frequency band below about 4 Hz (Langston 1979). The full data sets were published in Bianchi and Bokelmann (2018). Here we use the data from two stations deployed by the drilling site, that is KW01 and KW06, shown in Figures 2 and 3 as backazimuthal sweeps. The RFs obtained from the teleseismic events have been binned to increase the signal‐to‐noise ratio. Bins are obtained by the stacking of RFs for events occurring in the same backazimuth (±5°). The spatial filter used to define the events that belong to a single bin is 20° wide in backazimuth (baz) and 40° wide in Δ. The good backazimuthal coverage makes three‐dimensional structure modelling beneath the two stations possible from both the radial RF and transverse RF data sets (Figs 2a and 3a).

## 4 INVERSION METHOD

The one‐dimensional shear velocity model beneath the two stations has been investigated by Bianchi and Bokelmann (2018) who generated *a posteriori* probability‐density function of the *v_s_* at depth following the reversible‐jump Markov chain Monte Carlo (rjMcMC) approach developed by Piana Agostinetti and Malinverno (2010). In this paper, we use the recovered isotropic structure as a starting point for constraining a search for three‐dimensional (3D) features (i.e. dipping interfaces or/and anisotropic layers), through the neighbourhood algorithm (NA) search (Sambridge 1999). The rjMcMC search yields two main results: the Posterior Probablity Density (PPD) of the *S* velocity at depth and the distribution of the interface depth sampled during the chain; we used this information to build a parameter space for the following 3D *v_s_* modelling, and additional information from the transverse receiver function component, to give constraints on dipping interfaces and anisotropic layers. The mean *v_s_* model from the rjMcMC search was discretized with reference to the number and depth of the interfaces published in Bianchi and Bokelmann (2018). The *v_s_* models were divided into layers with uniform velocity and anisotropic parameters have been assigned to perform the NA search. According to previous information on the area, we set the possibility to explore the anisotropic parameters within the shallow unit (U1); this main layer is divided into three sublayers. The second layer in our parameter space has been set without anisotropy parameters (this would correspond to the metabasite depth, or the previously defined unit U2). The latter layer (or lower anisotropic layer) has been set with anisotropy, which would correspond to the lower (deeper) gneiss package (U3).

After some initial tentative, we select the parameter space that guarantees the best misfit reduction within a reasonable number of sampled models. To cross‐check that our parameter space does not bias our findings, we include in the Supporting Information Material additional results using different parameter spaces. The defined parameter space is shown in Table S1.

**Figure 4:**
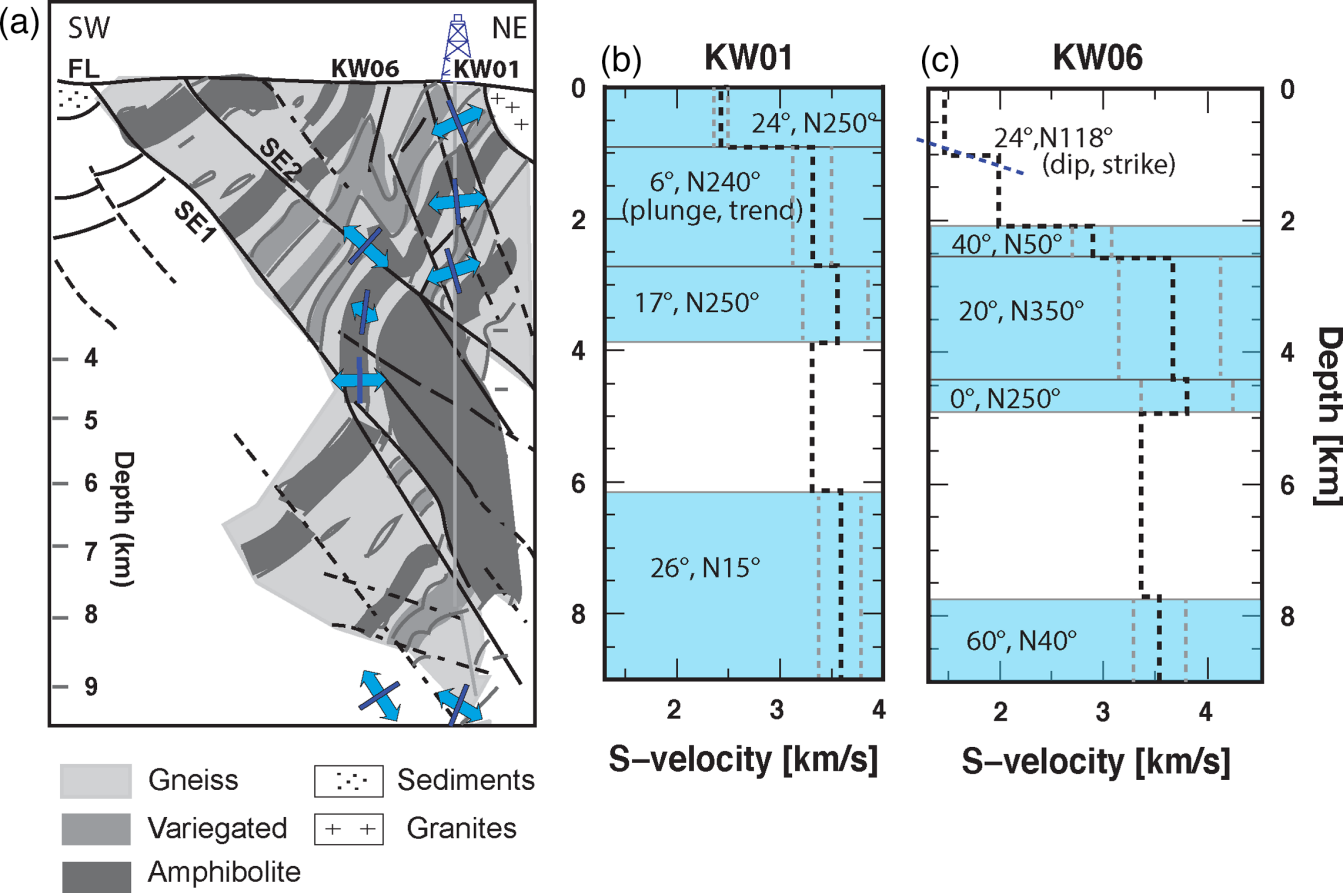
(a) The background is a schematic representation of the ZEV metamorphic body showing the location and depth of the drilling site with respect to the reconstructed structures of the metamorphic rocks (modified after Hirschmann 1996). Double‐sided arrows show the orientations of slow anisotropic axes projected along a SW–NE profile cutting the ZEV metamorphic body. Blue lines are drawn normal to the slow axes. The orientation of the blue lines is subparallel to the foliation planes for the lower layer and the upper layer below KW06, whereas it deviates for the upper layer below station KW01. Note the remarkable agreement of the inferred orientation of the foliation (blue lines) with geological features. (b) S‐wave velocity‐depth profile (black dashed line) for station KW01, blue area shows the depth location of anisotropic layers, grey dashed lines show the amount of anisotropy in each layer, plunge and trend angles of the anisotropy axes are specified. (c) Same as (b) for station KW06, blue dashed line shows the location of the inclined interface.

Modelling the receiver function (RF) is a classical inverse problem characterized by strong non‐uniqueness. Interfaces depths and S‐wave velocity, anisotropy percentage and plunge of the anisotropic symmetry axis display a clear trade‐off, which makes it difficult to draw simple quantitative inferences from the observations. The stochastic sampling used by the neighbourhood algorithm to explore the multidimensional parameter space for a range of acceptable velocity models uses the properties of Voronoi cells with the aim of finding an ensemble of models with acceptable data fit. We generated 1000 initial random samples inside the parameter space, and the 50 cells with the lowest misfit were resampled to produce 500 new samples. This process was repeated 500 times, for a total of 101,000 models explored for each station. We evaluate the standard deviation of the models parameters on the ensemble of models which generate synthetics that fit the data with 1.10 times lower misfit than the best‐fit model. The best fit model is then interpreted as representative of the ensemble. Synthetics are calculated using the RAYSUM code (Frederiksen and Bostock 2000) that models the propagation of a plane wave in dipping and anisotropic structures. Anisotropy was modelled as hexagonal with a unique axis of symmetry, which fits the characteristics of the transverse isotropy supported by laboratory experiments on the drill core samples (Kern *et al*. 1991). Here, we do not model multiple phases. To be coherent from many backazimuthal directions and thus recognizable in the RF patterns, multiple phases need to propagate through a homogeneous (anisotropic) structure covering a circle of about 10–15 km diameter around the station (approximately, for a 5 km depth target). From previous knowledge of the area (as clearly seen in Fig. 4a), we understand that the overall structure at the KTB drill site is not horizontally layered over such scale length; therefore, we can assume that the complex local structure prevents multiple phases to be coherently recorded in the RF signal.

### 4.1 Anisotropy model

In a hexagonal system, there is a single axis of symmetry; in the plane perpendicular to the axis of symmetry, every direction is indistinguishable. *P*‐wave propagation along the axis of symmetry can be either faster or slower than that in the perpendicular plane, corresponding to positive/negative anisotropy (Savage 1998). We opted for modelling anisotropy with slow symmetry axis due to the previous information on the Kontinental Tiefbohrung core samples, where the shear wave splitting has been recognized as most pronounced in the gneisses which hold a high percentage of mica. The mica as plate‐like mineral would have a slow propagation direction parallel to the [001] axis (Alexandrov and Rhyzova 1961; Kern and Schmidt 1990), which can be modelled by slow (negative) anisotropy (Levin and Park 1998). The other factor that may play a role is stress‐induced cracks; these would also be characterized by negative anisotropy. Focusing on negative anisotropy alone is not restrictive, because it has already been shown how fast and slow anisotropic axes reproduce the same pattern for opposite trend (e.g. Sherrington *et al*. 2004; Bianchi *et al*. 2008; Bianchi *et al*. 2010). In the considered models, the magnitude (percent) of *P* and *S* anisotropies are set to be equal to reduce the computation time. Dipping interfaces and dipping anisotropic layers produce similar signals that are difficult to distinguish (Savage 1998; Bianchi *et al*. 2008). Thus, we use the information about the location of anisotropic layers and the amount of anisotropy from previous studies (i.e. Berckhemer *et al*. 1997) to reproduce three‐dimensional features in the very shallow crust. In our parametrization, a plunge angle of 0° corresponds to a horizontal anisotropy symmetry axis, whereas a plunge angle of 90° corresponds to a vertical symmetry axis.

## 5 RESULTS

The output of the inversion method that we have employed here gives us the best‐fit model out of a family of models with 31 free parameters inverted. Twelve of the 31 free parameters are related to the anisotropic characteristics of the media, and describe trend and plunge of the symmetry axes, as well as the strength of anisotropy (in percent) of the four layers (three sublayers within the U1 + 1 layer in U3) of the velocity model bearing anisotropy.

We therefore focus here on the description of the anisotropy into the two main layers (U1 and U3) in the upper 10 km of the crust. For station KW01, three sublayers of the U1 have anisotropy with a slow axis trending to the SW, for each of these the plunge is less than 25° from the horizontal, and the percentage of anisotropy is increasing from the shallower to the deeper (from 5% to 8%). At 6.5 km depth, a deeper anisotropic layer is encountered (U3), which displays a symmetry axis trending to the NE. The counts of single iteration best‐fit values for the anisotropic layers (Fig. S1) are here shown for the robustness of the results. For station KW06, we run a first search using a two‐layered parameter space with one inclined interface, for constraining the high amplitudes observed within the first second of the receiver functions (RFs) (Fig. 3). The neighbourhood algorithm (NA) search performed returns an interface striking N118 ° at 1 km depth beneath the station, which is in agreement with the strike of the faults in the area and mapped by several previous works (e.g. Hirschmann 1996). Due to the large effect it has on the RF for that station, we have decided to use the subset of data in which the signals from the deeper layers have not been cancelled by the presence of the shallow dipping interface, and that is shown in Figure 3(b). Also for this station, the NA search finds anisotropy between 2 and 5 km depth, with increasing strength with depth (U1). Yet, the results give a less stable trend of the symmetry axis, probably caused by the imposed restriction of backazimuths and/or by the interference with the encountered dipping interface; at 7.5 km depth, the U3 is found, which shows an NE trend of its axis in agreement with the results for station KW01. Based on the family of the best‐fit models (ensemble of models which fit the observed wiggles with a misfit lower than 1.10 times the misfit of the best‐fit model), we give estimates on the uncertainties on the inverted parameters (Table 1). Errors on the thickness of the layers vary from ±0.1 km for the shallower layer to ±1.4 km for the lower layer; error on the amount of anisotropy is between ±1 and ±2%; for the trend of anisotropy, we find ±40° error estimate in the first and last layers; this value doubles for the second and third layers, showing the high variability of this result.

**Table 1:** **V**elocity model for KW01

		Thickness (km)	*v_s_* (km/s)	*v_p_*/*v_s_*	P‐S wave anisotropy (%)	Trend	Plunge	Strike	Dip
U1	Sublay1	0.9 ± 0.1	2.4	1.77	−5 ± 1	250 ± 40	24 ± 5		
	Sublay2	1.8 ± 0.3	3.3	1.76	−7 ± 2	240 ± 80	6 ± 3		
	Sublay3	1.2 ± 0.3	3.6	1.71	−8 ± 2	250 ± 70	17 ± 5		
U2		2.3 ± 0.4	3.3	1.73					
U3		7 ± 1	3.6	1.72	−5 ± 1	15 ± 40	26 ± 5		
		Halfspace	3.8	1.71				60	40

**Table 2:** Velocity model for KW06

		Thickness (km)	*v_s_* (km/s)	*v_p_*/*v_s_*	P‐S wave anisotropy (%)	Trend	Plunge	Strike	Dip
		1	1.4	1.63					
		1	1.9	1.73				118	24
**U1**	Sublay1	0.5 ± 0.4	2.9	1.79	−9 ± 3	50 ± 90	40 ± 10		
	Sublay2	1.8 ± 0.2	3.6	1.73	−13 ± 3	350 ± 90	20 ± 10		
	Sublay3	0.5 ± 0.4	3.8	1.75	−15 ± 3	250 ± 90	0 ± 10		
U2		2.8 ± 0.5	3.3	1.70					
**U3**		4.5 ± 0.5	3.5	1.73	−7 ± 2	40 ± 70	60 ± 10		
		Halfspace	3.7	1.76				314	30

To verify that the choice of our parameter space does not bias the solution, we tested the presence of anisotropy in the layer U2. The results converge towards anisotropy with a nearly vertical axis (plunge equals to 75° ± 8°, Fig. S2), thus we exclude the presence of anisotropy there because such a geometry does not generate any relevant signal on the T component of the RF. By enlarging the values of anisotropy magnitude (0 to −15%) and plunge (0° to 90°) for the layers in U1 and U3, we show that the choice of the boundaries of our parameter space does not affect the results of the NA search. In this case, we include models without anisotropy (or with limited effects on T‐RF patterns, that is plunge angles larger than 60°). The results are shown in Fig. S3. Best‐fit and standard deviation values highlight that a strong anisotropy is still present in layers U1 and U3 (between −5% and −9%), and that the plunge of the symmetry axes are not close to vertical.

## 6 DISCUSSION

We have determined the anisotropic parameters for a sequence of layers under two seismic stations near the Kontinental Tiefbohrung (KTB) borehole, and we will later compare these in detail with the subsurface structure which is known from the KTB borehole.

Lithological changes with depth, changes in the fabric orientation, presence of zones of mechanical weakness associated with lower seismic velocities as cracks or cataclastic fracture zones are all factors which contribute to cause anisotropy. We schematize here the main information regarding these points from the wide literature produced by previous studies at the KTB site.

1. The lithological units of the zone of Erbendorf‐Vohenstrauss (ZEV) extend down to the final depth of the borehole and are divided into paragneiss, amphibolites and variegated (alternating sequences of gneiss and amphibolites) (Fig. 1) (Emmermann and Lauterjung 1997). The whole profile can be subdivided into three main uniform units: (U1) consisting of mainly paragneiss (0 to 3.2 km depth); (U2) composed of metabasites, mainly amphibolite, metagabbros and metabasalt (3.2 to 7.3 km depth); and (U3) made of mainly paragneiss (7.3 km depth until the bottom of the borehole, 9.1 km depth) (Berckhemer *et al*. 1997).
2. The metamorphic rocks show a steep dip between 60° to 80° towards SW or NE and are strongly folded with subhorizontal axial planes and NNW–SSE trending fold axes (e.g. Duyster *et al*. 1995; Hirschmann and Lapp 1995). The KTB depth profile has been subdivided into three structural units with homogeneous dip: (U1) mainly SW and minor NE dip, (U2) mainly E and minor W dip and (U3) SW dip (Berckhemer *et al*. 1997). The local fault system is associated to the Franconian Lineament (FL) which strikes N140° at about 5 km SW from the KTB borehole (Fig. 1).
3. The seismic reflectors named SE1 and SE2 are recognized to be cataclastic zones related to the local fault system and related to the FL (Hirschmann 1996; Hluchy, Körbe and Thomas 1992), outcropping on surface at about 4.5 km distance from KW01. Considering an average dip of 55° to the NE of the faults, we would encounter the SE1 at 6.4 km and at 3.5 km beneath KW01 and KW06, respectively; the estimated depth of SE2 is at about 3.5 km and 2.5 km for stations KW01 and KW06, respectively. These two also mainly correspond to the boundaries between the three lithologically uniform units below station KW01.
4. Stress measurements in the pilot hole (Baumgärtner *et al*. 1990; Mastin *et al*. 1991) show an NNW–SSE oriented direction of maximum horizontal principal stress and strike‐slip to normal faulting stress magnitudes. The wave velocities at the KTB drill site are affected by the presence of open microcracks until a critical depth of 5–6 km (Kern *et al*. 1991); according to the same author, in the mica‐rich genisses, the oriented microfractures are occurring parallel to the morphological sheet planes.
5. A fast anisotropic axis (for both compressional and shear waves) has been detected that is oriented NW–SE parallel to the foliation planes of the metamorphic body (Hirschmann 1996; Berckhemer *et al*. 1997; Harjes *et al*. 1997). Based on array data in a similar setting (GERESS array), Bokelmann (1995) had also found a good agreement of the orientation of fast planes with the known foliation orientation of rocks in the area (based on P‐wave polarization). Laboratory measurements on rock samples had predicted a considerable amount of anisotropy (Stroh *et al*. 1990; Kern *et al*. 1991; Siegesmund *et al*. 1993). Lüschen *et al*. (1991, 1996) and Hirschmann (1994) proved the existence of split shear waves with an observed velocity difference of up to 10%, locally even as high as 14% (Rabbel *et al*. 2004).

To compare with rock fabrics, we inspect the retrieved trend and plunge angles of the slow anisotropic axes, and project them on the SW–NE cross section of the ZEV summarizing the main orientation of the foliation and of the faults (Hirschmann 1996) (Fig. 4a). For the deeper anisotropic layer found at both stations, the inferred NE‐trend of the slow anisotropic axis corresponds to the minimum velocity of the seismic wave, which is perpendicular to the rock foliation in mica‐rich rocks such as the gneiss of the ZEV. We know from literature that the foliation planes are dipping to the SW; that would therefore fit to a symmetry axis of the mica plunging towards the NE. We observe therefore a perfect fit between the true foliation dipping to SW (shown in the background of Fig. 4a) and the foliation obtained by the inferred anisotropy symmetry axis (blue lines, Fig. 4a); we therefore attribute the anisotropy as caused by textural velocity. The inferred NE‐trend of the slow axis is also perpendicular to the NW–SE fast anisotropy axis from previous studies (e.g. Bopp 1992; Hirschmann 1996; Rabbel *et al*. 2004). In the shallower layer, we may also be dealing with open cracks that would be expected to be aligned with the stress field up to several kilometres depth (Kern *et al*. 1991); we know that the tectonic stress in the vicinity of the KTB is expected to have an orientation of (N150°E) (Zoback *et al*. 1993). All three factors potentially causing seismic anisotropy (fabric, faults and σ_H_‐aligned cracks) are striking in NW–SE orientation, but faulting and foliation dip towards opposite directions and affecting the wave propagation; we argue that the causes of the deviation from being parallel to the texture are due to the combined influence of faults and cracks.

The striking finding of our analysis is the perfect match between the depth and thickness of the mica‐rich gneiss found in the core‐log and the depth extent of the inferred anisotropic U1 and U3 layers. Figure 4(a) shows that receiver function (RF) data constrain anisotropy within the gneiss layers, avoiding the amphibolite layer even if the parameter space (Table S1) gives a potential thickness for U1 between 1 and 5 km and a potential boundary between U2 and U3 between 2 and 8 km. Locating anisotropy at depth is a difficult task for exploration geophysics (e.g. repeated Amplitude Versus Offset (AVO) is a common but expensive tool (Rüger 2001)). It is of particular interest that we can demonstrate here a relatively cheap method able to locate the anisotropic rock volumes at depth. Accounting for anisotropy has strong implication for reservoir characterization, because its presence might cause significant problems in the interpretation of active seismic data and therefore on the properties of investigated depth structures (e.g. Asaka 2018). We have seen that RFs can provide important information on the subsurface location and character of seismic anisotropy from passively recorded data. The application is computationally efficient, and it may in principle be of interest also for industrial (e.g. oil/gas) applications, as well as underground storage, although it will in general not yield a resolution comparable with that of reflection data (e.g. Tsvankin *et al*. 2010), due to the longer wavelengths.

## 7 CONCLUSIONS

With the application of the receiver function (RF), a passive seismological technique, in the Kontinental Tiefbohrung (KTB) area in Southern Germany, we have acquired some unique proof of the direct correspondence between *in situ* rock texture and seismic anisotropy.

We gain information below two seismic stations located nearby the KTB drilling hole, where we verify the presence of *in situ* anisotropy; in particular, we infer the presence of two distinct anisotropic layers within the upper crust. The depth of these two layers corresponds to the location of mica‐rich paragneiss which show intense foliation, this let us gain larger information with respect to previous *in situ* studies where anisotropy was identified as due to the entire Zone of Erbendorf‐Vohenstrauss (ZEV) thickness. We infer higher anisotropic percentages at shallower depths probably related to the presence of cracks. Concerning the causes of the seismic anisotropy, we conclude that for the lower anisotropic layer the symmetry axis of the slow shear‐wave is perpendicular to the main foliation of the paragneiss, and is ascribed as due to the alignment of the minerals (micas), whereas for the upper anisotropic layer, the slow anisotropic axis deviates from being perpendicular to the foliation plane, probably due to the presence of intense faulting and of the crack system. Moreover, RF can provide important information on the depth location of seismic anisotropy, and can be used as a complementary or explorative tool for underground exploitation, due to its computational efficiency and low cost.

## Figures and Tables

**Figure A1:**
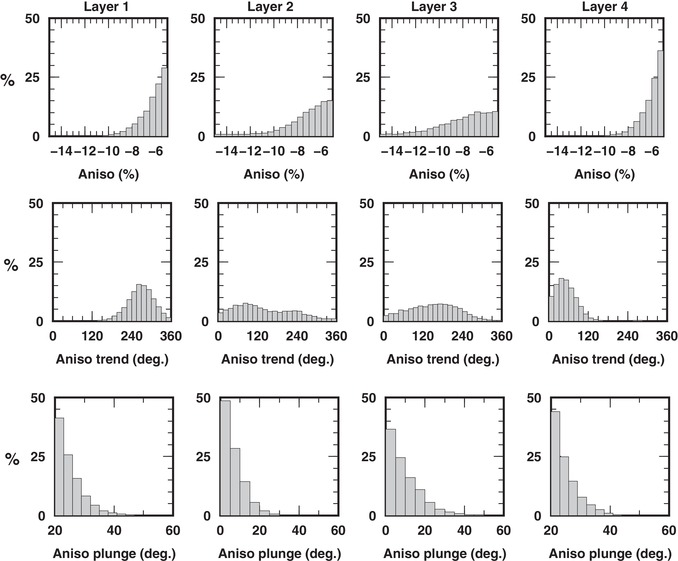
Count of single‐iteration best‐fit values for anisotropy concerning anisotropy strength, trend (from N) and plunge (from horizontal) (respectively, upper, middle and bottom row), for the four anisotropic layers modelled for station KW01.

**Figure A2:**
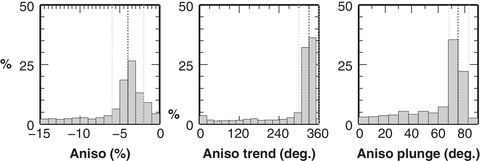
Count of single‐iteration best‐fit values for anisotropy concerning anisotropy strength, trend (from N) and plunge (from horizontal) for the layer U2, black dashed line shows the value of the best‐fit model, and grey dashed lines show the standard deviation.

**Figure A3:**
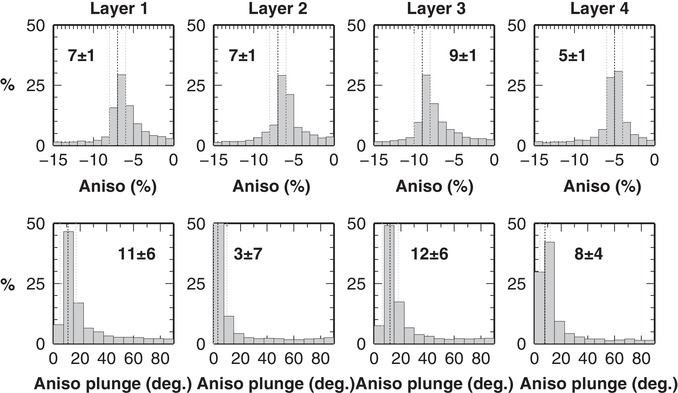
Count of single‐iteration best‐fit values for anisotropy concerning anisotropy strength, and plunge (from horizontal) for the layers in U1 and U3 considering an unconstrained parameter space. Black dashed line shows the value of the best‐fit model, and grey dashed lines show the standard deviation, these values are also reported in each panel.

**Figure A4:**
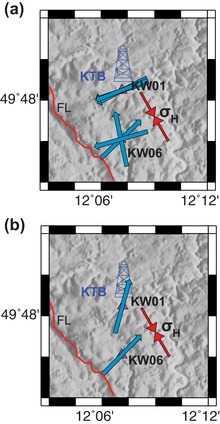
a) and b) Map view showing the trend of anisotropic axes (in (a) the upper layer, and in (b) the lower layer) in relation with the maximum horizontal stress (*σ_H_*) and the surface trace of the FL.

## APPENDIX 1

### 1.1

**Table A1:** Model min/max defining the parameter space for the NA search

KW01							
Thickness (km)	Vs (km/s)	Vp/Vs	P‐S wave anisotropy (%)	Trend	Plunge	Strike	Dip
0.2/1.0	2/3	1.70/1.79	−5/−15	0/360	20/60		
0.5/2.0	3.3/3.8	1.70/1.79	−5/−15	0/360	0/60		
0.3/2.0	3.5/3.8	1.70/1.79	−5/−15	0/360	0/60		
1.0/3.0	3.3/3.8	1.70/1.79					
2.0/8.0	3.5/3.8	1.70/1.79	−5/−15	0/360	20/60		
halfspace	3.7/4.0	1.70/1.79				0/360	30/70
KW06							
1	1.4	1.63					
0.5/2.5	2.0	1.73				118	24
0.2/1.0	2/3	1.70/1.79	−5/−15	0/360	20/60		
0.5/2.0	3.3/3.8	1.70/1.79	−5/−15	0/360	0/60		
0.3/2.0	3.5/3.8	1.70/1.79	−5/−15	0/360	0/60		
1.0/3.0	3.3/3.8	1.70/1.79					
2.0/8.0	3.5/3.8	1.70/1.79	−5/−15	0/360	20/60		
halfspace	3.7/4.0	1.70/1.79				0/360	30/70

**Table:**

#DATE	OT	LON	LAT	M
2012/07/12	12:51:58	45.45	151.66	5.8
2012/07/20	06:10:25	49.41	155.91	6
2012/07/20	06:32:56	49.35	156.13	5.8
2012/07/25	00:27:45	2.71	96.04	6.4
2012/07/26	05:33:33	−17.59	66.39	6.7
2012/07/29	09:20:55	47.38	139.07	5.6
2012/07/29	12:22:11	14.19	−92.29	5.9
2012/08/02	09:38:30	−8.41	−74.26	6.1
2012/08/10	18:37:43	52.63	−167.42	6.2
2012/08/14	02:59:38	49.8	145.06	7.7
2012/08/18	09:41:52	−1.32	120.1	6.3
2012/08/21	17:39:38	−0.17	92.06	5.6
2012/08/25	14:16:17	42.42	142.91	5.9
2012/08/26	15:05:37	2.19	126.84	6.6
2012/08/27	04:37:19	12.14	−88.59	7.3
2012/08/29	19:05:11	38.42	141.81	5.5
2012/08/31	12:47:33	10.81	126.64	7.6
2012/08/31	23:37:58	10.39	126.72	5.6
2012/11/22	13:07:10	−22.8	−63.69	5.8
2012/11/28	03:09:49	−4.5	−76.12	5.6
2012/12/01	08:00:57	58.67	−154.44	5.7
2012/12/07	08:18:23	37.92	144.02	7.3
2012/12/07	22:59:57	37.77	143.78	5.6
2012/12/14	10:36:01	31.19	−119.6	6.4
2012/12/15	04:49:29	52.27	174.06	6
2013/01/05	08:58:19	55.28	−134.67	7.5
2013/01/08	07:51:31	40.29	142.22	5.5
2013/01/21	22:22:51	4.98	95.97	6
2013/02/02	14:17:34	42.79	143.17	6.9
2013/02/09	14:16:07	1.17	−77.41	7
2013/02/13	03:57:47	30.31	131.4	5.5
2013/02/16	04:37:35	5.96	125.85	6.2
2013/02/22	12:01:59	−27.89	−63.18	6.1
2013/02/25	07:23:56	36.9	139.24	5.7
2013/02/28	14:05:52	50.92	157.36	6.8
2013/03/01	12:53:54	50.97	157.56	6.3
2013/03/01	13:20:52	50.92	157.53	6.5
2013/03/07	03:36:46	24.31	121.49	5.5
2013/03/09	14:56:29	50.89	157.29	5.8
2013/03/24	04:18:34	50.72	160.19	6
2013/03/25	23:02:14	14.7	−90.32	6.2
2013/03/27	02:03:20	23.84	121.13	6
2013/03/31	07:46:18	39.12	141.88	5.5
2013/04/01	18:53:17	39.58	143.1	6
2013/04/03	16:35:46	19.3	95.72	5.6
2013/04/05	13:00:01	42.8	131.08	6.2
2013/04/06	00:29:55	42.79	130.93	5.8
2013/04/10	20:20:27	20.8	122.07	6
2013/04/12	20:33:17	34.4	134.84	5.8
2013/04/17	08:57:24	34.18	139.46	5.7
2013/04/17	12:03:31	38.53	141.56	5.9
2013/04/19	03:05:52	46.26	150.85	7.2
2013/04/19	19:58:40	50.07	157.45	6.1
2013/04/20	13:12:52	50.12	157.13	6.1
2013/04/20	13:18:08	50.03	157.43	5.6
2013/04/21	03:22:17	29.96	138.96	6.2
2013/04/22	01:16:38	18.23	−102.05	6.1
2013/04/29	13:01:42	35.76	140.97	5.5
2013/05/10	19:56:05	−29.16	−13.21	5.8
2013/05/12	07:30:03	13.62	−91.34	5.8
2013/05/12	20:06:45	52.56	−171.98	5.7
2013/05/12	22:42:45	43.98	147.85	5.6
2013/05/14	00:32:26	18.82	145.26	6.8
2013/05/18	04:05:44	−53.16	22.19	5.7
2013/05/18	05:48:01	37.79	141.51	6
2013/05/19	18:44:09	52.29	160.18	5.9
2013/05/19	22:40:28	52.34	159.76	5.7
2013/05/20	23:01:25	52.43	160.24	5.6
2013/05/21	01:55:04	52.44	160.48	6
2013/05/21	03:05:50	52.4	160.38	5.8
2013/05/21	03:08:22	52.43	160.02	5.8
2013/05/21	04:59:37	52.28	160.19	5.6
2013/05/21	05:43:21	52.34	160.01	6.1
2013/05/21	08:25:54	23.5	123.73	5.5
2013/05/21	14:51:19	52.57	160.65	5.6
2013/05/24	05:44:48	54.91	153.34	8.3
2013/05/24	14:56:31	52.26	151.54	6.7
2013/05/27	20:22:04	52.33	160.15	5.5
2013/06/02	05:43:04	23.79	121.08	6.2
2013/06/04	11:00:07	45.44	150.95	5.5
2013/06/04	11:01:57	45.4	151	5.5
2013/06/07	16:38:04	24.08	122.57	5.7
2013/06/13	16:47:23	−9.96	107.27	6.7
2013/06/15	17:34:28	11.52	−87.2	6.5
2013/06/16	05:19:02	18.36	−99.02	6.1
2013/06/27	08:38:09	1.16	127.17	5.7
2013/06/28	23:51:48	24.08	122.36	5.5
2013/07/02	07:37:03	4.72	96.57	6.1
2013/07/03	03:37:32	51.43	−166.86	5.6
2013/07/03	03:40:26	51.51	−167.01	5.7
2013/07/06	05:05:08	−3.26	100.53	6
2013/07/16	14:09:27	43.06	145.45	5.5
2013/07/17	02:37:43	−15.64	−71.81	6
2013/07/20	06:06:23	36.21	141.82	5.6
2013/07/22	07:01:42	−46.16	34.85	6.3
2013/08/04	03:28:51	38.26	141.81	5.8
2013/08/04	15:56:34	47.02	145.3	5.8
2013/08/12	09:49:32	−5.43	−81.97	6.2
2013/08/13	15:43:14	5.78	−78.23	6.6
2013/08/17	16:32:31	−34.92	54	6.1
2013/08/21	12:38:32	17.06	−99.34	6.2
2013/09/05	12:29:18	10.63	−86.07	5.9
2013/09/07	00:19:00	14.77	−92.01	6.5
2013/09/14	15:42:44	51.61	−174.75	6
2013/09/15	16:21:39	51.69	−174.74	6.2
2013/09/25	13:58:15	52.94	171.3	5.5
2013/09/25	16:42:43	−15.91	−74.63	7
2013/09/26	06:46:05	14.45	−93.36	5.7
2013/10/01	03:38:21	53.17	152.88	6.7
2013/10/12	02:10:29	10.86	−62.37	6
2013/10/13	17:32:46	4.04	95.96	5.6
2013/10/15	00:12:33	9.92	124.1	7.1
2013/10/19	17:54:58	26.37	−110.17	6.5
2013/10/24	17:57:35	14.28	93.16	5.5
2013/10/25	17:10:17	37.22	144.69	7.1
2013/10/27	18:13:06	37.21	144.62	5.6
2013/10/31	12:02:08	23.57	121.47	6.3
2013/11/09	22:37:51	35.95	140.03	5.6
2013/11/12	07:03:51	54.81	162.09	6.4
2013/11/19	13:32:55	2.71	128.43	6.1
2013/11/25	05:56:50	45.65	151.03	5.9
2013/12/07	07:36:26	56.85	−155.22	5.7
2013/12/08	17:24:54	44.5	149.17	6.1
2014/01/16	07:33:11	51.33	−179.19	5.8
2014/02/18	23:35:58	−14.23	−75.72	5.9
2014/02/26	21:13:39	53.7	−171.86	6.1
2014/03/02	09:37:55	12.59	−87.63	6.2
2014/03/02	20:11:22	27.47	127.32	6.5
2014/03/10	05:18:13	40.73	−124.97	6.9
2014/03/13	13:20:58	51.36	−179.29	5.5
2014/03/13	17:06:50	33.73	131.67	6.3
2014/03/16	21:16:29	−19.86	−70.58	6.7
2014/03/17	05:11:33	−19.99	−70.88	6.2
2014/03/19	12:19:27	24.07	122.33	5.7
2014/03/21	13:41:08	7.69	94.2	6.4
2014/04/01	23:46:47	−19.68	−70.85	8.1
2014/04/02	16:13:28	7.96	−82.37	6
2014/04/02	23:22:48	39.2	141.77	5.5
2014/04/03	01:58:29	−20.28	−70.51	6.5
2014/04/03	02:43:18	−20.43	−70.3	7.6
2014/04/04	01:37:50	−20.61	−70.62	6.2
2014/04/10	23:27:46	12.54	−86.36	6.1
2014/04/11	20:29:15	11.72	−85.98	6.6
2014/04/15	03:57:02	−53.72	8.68	6.8
2014/04/18	14:27:26	17.46	−100.87	7.2
2014/04/21	20:45:23	17.42	120.07	5.5
2014/04/24	03:10:12	49.78	−127.49	6.5
2014/04/28	00:43:51	19.71	120.12	5.5
2014/05/02	09:15:19	37.89	144.26	5.6
2014/05/04	20:18:24	34.84	139.36	6
2014/05/05	11:08:45	19.68	99.78	6.2
2014/05/08	17:00:17	17.33	−100.75	6.4
2014/05/10	07:36:01	17.28	−100.73	6.1
2014/05/13	06:35:24	7.18	−82.37	6.5
2014/05/15	10:16:43	9.41	122.14	6.2
2014/05/18	01:02:28	4.31	92.73	6
2014/05/21	00:21:13	23.71	121.56	5.5
2014/05/21	10:06:15	17.19	−94.88	5.8
2014/05/28	21:15:05	18.13	−68.39	5.8
2014/06/01	10:07:11	2.02	89.78	5.8
2014/06/14	11:11:01	−10.02	91.02	6.5
2014/06/14	17:31:42	39.47	141.04	5.6
2014/06/15	18:19:13	36.65	141.72	5.6
2014/06/15	20:14:51	37.17	141.01	5.6
2014/06/16	06:39:32	1.64	−79.35	5.6
2014/06/23	20:53:09	51.85	178.76	7.9
2014/06/24	03:15:40	52.26	176.87	6.6
2014/06/24	08:12:32	52.28	176.59	5.7
2014/06/29	05:56:30	24.39	142.64	6.2
2014/06/30	19:55:33	28.39	138.9	6.2
2014/07/03	02:56:38	55.31	166.84	5.7
2014/07/03	12:05:22	55.27	166.97	5.8
2014/07/04	22:42:04	39.72	142.04	5.7
2014/07/05	09:39:31	2.03	97.08	6
2014/07/07	11:23:58	14.88	−92.3	6.9
2014/07/08	09:05:23	42.7	141.47	5.5
2014/07/11	19:21:59	37.06	142.54	6.5
2014/07/14	08:00:01	5.75	126.57	6.3

## References

[gpr12883-bib-0001] Alexandrov K.S. and Rhyzova T.J. 1961 Elastic properties of the rock‐forming minerals: layered silicates. Bull. Acad. Sci. U.S.S.R. Geophys. Ser. 9, 1165–1168.

[gpr12883-bib-0002] Almqvist B.S.G. and Mainprice D. 2017 Seismic properties and anisotropy of the continental crust: predictions based on mineral texture and rock microstructure. Reviews of Geophysics 55, 367–433.

[gpr12883-bib-0003] Ammon C.J. 1991 The isolation of receiver effects from teleseismic P waveforms. Bulletin of the Seismological Society of America 81, 2504–2510.

[gpr12883-bib-0004] Ammon C.J. , Randall G.E. and Zandt G. 1990 On the non‐uniqueness of receiver function inversions. Journal of Geophysical Research 95, 15303–15318.

[gpr12883-bib-0005] Anderson D.L. , Minster B. and Cole N. 1974 The effect of oriented cracks on seismic velocities. Journal of Geophysical Research 79, 4011–4015.

[gpr12883-bib-0006] Asaka M. 2018 Anisotropic AVO: implications for reservoir characterization. The Leading Edge 37, 916–923.

[gpr12883-bib-0007] Audet P. 2015 Layered crustal anisotropy around the San Andreas Fault near Parkfield, California. Journal of Geophysical Research‐Solid Earth 120, 3527–3543.

[gpr12883-bib-0008] Babuška V. and Cara M. 1991 Seismic Anisotropy in the Earth. London, UK: Kluwer Academic Publishers.

[gpr12883-bib-0009] Barruol G. and Mainprice D. 1993 3‐D seismic velocities calculated from lattice‐preferred orientation and reflectivity of a lower crustal section: examples of the Val Sesia section (Ivrea zone, northern Italy). Geophysical Journal International 115, 1169–1188.

[gpr12883-bib-0011] Berckhemer H. , Rauen A. , Winter H. , Kern H. , Kontny A. , Lienert M. *et al* 1997 Petrophysical properties of the 9‐km‐deep crustal section at KTB. Journal of Geophysical Research 102, 18337–18361.

[gpr12883-bib-0012] Bianchi I. , Anselmi M. , Apoloner M.T. , Qorbani E. , Gribovski K. and Bokelmann, G. 2015a The installation campaign of 9 seismic stations around the KTB site to test anisotropy detection by the receiver function technique. Advances in Geosciences 41, 11–23.

[gpr12883-bib-0013] Bianchi I. and Bokelmann G. 2018 Imaging the Variscan suture at the KTB deep drilling site, Germany. Geophysical Journal International 213, 2138–2146.

[gpr12883-bib-0014] Bianchi I. , Bokelmann G. and Shiomi K. 2015b Crustal anisotropy across northern Japan from receiver functions. Journal of Geophysical Research: Solid Earth 120, 4998–5012. 27478718 10.1002/2014JB011681 PMC4949574

[gpr12883-bib-0015] Bianchi I. , Lucente F.P. , Di Bona M. , Govoni A. and Piana Agostinetti N. 2016 Crustal structure and deformation across a mature slab tear zone: the case of southern Tyrrhenian Subduction (Italy). Geophysical Research Letters 43, 12380–12388.

[gpr12883-bib-0016] Bianchi I. , Park J. , Piana Agostinetti N. and Levin V. 2010 Mapping seismic anisotropy using harmonic decomposition of Receiver Functions: an application to Northern Apennines, Italy. Journal of Geophysical Research, 115, B12317.

[gpr12883-bib-0017] Bianchi I. , Piana Agostinetti N. , De Gori P. and Chiarabba C. 2008 Deep structure of the Colli Albani Volcanic District (central Italy) from Receiver Function analysis. Journal of Geophysical Research 113, B09313.

[gpr12883-bib-0018] Bokelmann G.H.R. 1995 P‐wave array polarization analysis and effective anisotropy of the brittle crust. Geophysical Journal International 120, 145–162.

[gpr12883-bib-0019] Bopp M. 1992 Shear‐wave splitting observed by wide‐angle measurement. KTB Report *92–5*, pp. 297–308.

[gpr12883-bib-0020] Bostock M.G. and Christensen N.I. 2012 Split from slip and schist: crustal anisotropy beneath northern Cascadia from non‐volcanic tremor. Journal of Geophysical Research 117, B08303.

[gpr12883-bib-0021] Brudy M. , Zoback M.D. , Fuchs F. , Rummel F. and Baumgärtner J. 1997 Estimation of the complete stress tensor to 8 km depth in the KTB scientific drill holes: implications for crustal strength. Journal of Geophysical Research 102, 18453–18457.

[gpr12883-bib-0022] Christensen N.I. 1965 Measurements of dynamic properties of rock at elevated temperatures and pressures In: Measurements of Rock Properties at Elevated Pressures and Temperatures (eds Pincus H.J. and Hoskins E.R.), pp. 93–107. West Conshohocken, PA: ASTM International.

[gpr12883-bib-0023] Christensen N.I. and Mooney W.D. 1995 Seismic velocity structure and composition of the continental crust: a global review. Journal of Geophysical Research 100, 9761–9788.

[gpr12883-bib-0024] DEKORP and Orogenic processes Working Groups . 1999 Exhumation of subducted crust— the Saxonian granulites from reflection seismic experiment GRANU’ 95. Tectonics 18, 756–773.

[gpr12883-bib-0025] DEKORP Research Group . 1987 Near‐vertical and wide‐angle seismic surveys in the Black Forest, SW Germany. Journal of Geophysical 62, 1–30.

[gpr12883-bib-0026] DEKORP Research Group . 1988 Results of the DEKORP 4/KTB Oberpfalz deep seismic reflection investigations. Journal of Geophysical 62, 69–101.

[gpr12883-bib-0027] Duyster J. , Kontny A. , de Wall H. and Zulauf G. 1995 Postvariszische Krustenstapelung am Westrand der Böhmischen Masse. Geowissenschaften 134, 135–141.

[gpr12883-bib-0028] Eckhardt C. and Rabbel W. 2011 *P*‐receiver functions of anisotropic continental crust: a hierarchic catalogue of crustal models and azimuthal waveform patterns. Geophysical Journal International, 187, 439–479.

[gpr12883-bib-0029] Eisbacher G.‐H. , Lueschen E. and Wickert F. 1989 Crustal‐scale thrusting and extension in the Hercynian Schwarzwald and Vosges, Central Europe. Tectonics 8, 1–21.

[gpr12883-bib-0030] Emmermann R. 1989 The KTB pilot hole: tectonic setting, technical data and first results In: The German Continental Deep Drilling Program(KTB): Site‐selection Studies in the Oberpfalz and Schwarzwald (eds Emmermann R. and Wohlenberg J.), pp. 527–553. Berlin–Heidelberg, Germany: Springer.

[gpr12883-bib-0073] Emmermann R. and Lauterjung J. 1997 The German Continental Deep Drilling Program KTB: Overview and major results. J. Geophys. Res. 102, 18179–18201, 10.1029/96JB03945.

[gpr12883-bib-0031] Frederiksen A.W. and Bostock M.G. 2000 Modelling teleseismic waves in dipping anisotropic structures. Geophysical Journal International 141, 401–412.

[gpr12883-bib-0032] Girardin N. and Farra V. 1998 Azimuthal anisotropy in the upper mantle from observation of P‐to‐S converted phases: application to southeast Australia. Geophysical Journal International 133, 615–629.

[gpr12883-bib-0033] Haak V. and Jones A. 1997 Introduction to special section: the KTB deep drill hole. Journal of Geophysical Research 102, 175–177.

[gpr12883-bib-0034] Harjes H.P. , Bram K. , Dürbaum H.‐J. , Gebrande H. , Hirschmann G. , Janik M. , *et al* 1997 Origin and nature of crustal reflections: results from integrated seismic measurements at the KTB superdeep drilling site. Journal of Geophysical Research 102, 18267–18288.

[gpr12883-bib-0035] Hirschmann G. 1996 KTB—the structure of a Variscan terrane boundary: seismic investigation—drilling—models. Tectonophysics 264, 327–339.

[gpr12883-bib-0036] Hirschmann G. and Lapp M. 1995 Evaluation of the structural geology of the KTB Hauptbohrung (KTB‐Oberpfalz HB) *KTB Report 94‐1*, pp. 285–308. Hannover, Germany: Niedersächsisches Landesamt für Bodenforsch.

[gpr12883-bib-0037] Hluchy P. , Körbe M. and Thomas R. 1992 Preliminary interpretation of the 3D‐seismics survey at the KTB location *KTB Report 92‐5*, pp. 31–52. Hannover, Germany: Niedersächsisches Landesamt für Bodenforsch.

[gpr12883-bib-0038] Kern H. , Popp T. and Schmidt R. 1994 The effect of a deviatoric stress on physical rock properties. Surveys in Geophysics 15, 467.

[gpr12883-bib-0039] Kern H. and Schmidt R. 1990 Physical properties of KTB core samples at simulated in‐situ conditions. Scientific Drilling 1, 217–223.

[gpr12883-bib-0040] Kern H. , Schmidt R. and Popp T. 1991 The velocity and density structure of the 4000 m crustal segment at the KTB drilling site and their relationship to lithological and microstructural characteristics of the rocks: an experimental approach. Scientific Drilling 2, 130–145.

[gpr12883-bib-0041] Langston C.A. 1979 Structure under Mount Rainier, Washington, inferred from teleseismic body waves. Journal of Geophysical Research 84, 4749–4762.

[gpr12883-bib-0042] Levin V. and Park J. 1998 P‐SH conversions in layered media with hexagonally symmetric anisotropy: a cookbook. Pure and Applied Geophysics 151, 669–697.

[gpr12883-bib-0043] Licciardi A. , Eken T. , Taymaz T. , Agostinetti N.P. and Yolsal‐Çevikbilen S. 2018 Seismic anisotropy in central North Anatolian Fault Zone and its implications on crustal deformation. Physics of the Earth and Planetary Interiors 277, 99–112.

[gpr12883-bib-0044] Lloyd G.E. , Butler R.W. , Casey M. and Mainprice D. 2009 Mica, deformation fabrics and the seismic properties of the continental crust. Earth and Planetary Science Letters 288, 320–328.

[gpr12883-bib-0045] Lüschen E. , Bram K. , Söllner W. and Sobolev S. 1996 Nature of seismic reflections and velocities from VSP‐experiments and borehole measurements at the KTB deep drilling site in southeast Germany. Tectonophysics 264, 309–326.

[gpr12883-bib-0046] Lüschen E. , Soellner W. , Hohrath A. and Rabbel W. 1991 Integrated P‐ and S‐wave borehole experiments at the KTB deep drilling site in the Oberpfalz area (SE Germany) Continental Lithosphere: Deep Seismic Reflections, Vol. 22 (eds Meissner R., Brown L., Dürbaum H.‐J., Franke W., Fuchs K. and Seifert F.), pp. 121–133. Washington, DC: AGU.

[gpr12883-bib-0047] Mastin L.G. , Heinemann B. , Krammer A. , Fuchs K. and Zoback M.D. 1991 Stress orientation in the KTB pilot hole determined from wellbore breakouts. Scientific Drilling 2, 1–12.

[gpr12883-bib-0048] Muller J. , Janik M. and Harjes H.‐P. 1999 The use of wave‐field directivity for velocity estimation: moving source profiling (MSP) experiments at KTB. Pure and Applied Geophysics 156, 303–318.

[gpr12883-bib-0049] O'Brien P.J. , Duyster J. , Grauert B. , Schreyer W. , Stoeckhert W. and Weber K. 1997 Crustal evolution of the KTB drill site: from old‐est relics to the late Hercynian granites. Journal of Geophysical Research‐Solid Earth 102, 18203–18220.

[gpr12883-bib-0050] Okaya D. , Christensen N.I. , Ross Z. and Wu F. 2016 Terrane‐controlled crustal shear wave splitting in Taiwan. Geophysical Research Letters 43, 556–563.

[gpr12883-bib-0051] Okaya D. , Rabbel W. , Beilecke T. and Hasenclever J. 2004 P wave material anisotropy of tectono‐metaorphic terrane: an active source seismic experiment at the KTB super‐deep drill hole, southeast Germany. Geophysical Research Letters 31, L24620.

[gpr12883-bib-0052] Okaya D. , Vel S.S. , Song W.J. and Johnson S.E. 2019 Modification of crustal seismic anisotropy by geological structures (“structural geometric anisotropy”). Geosphere 15, 146–170.

[gpr12883-bib-0053] Ozacar A. and Zandt G. 2004 Crustal seismic anisotropy in central Tibet: implications for deformational style and flow in the crust. Geophysical Research Letters 31, L23601.

[gpr12883-bib-0054] Park J. and Levin V. 2000 Receiver functions from multiple‐taper spectral correlation estimates. Bulletin of the Seismological Society of America 90, 1507–1520.

[gpr12883-bib-0055] Piana Agostinetti N. , Bianchi I. , Amato A. and Chiarabba C. 2011 Fluid migration in continental subduction. Earth and Planetary Science Letters 302, 267–278.

[gpr12883-bib-0056] Piana Agostinetti N. and Malinverno A. 2010 Receiver function inversion by trans‐dimensional Monte Carlo sampling. Geophysical Journal International 181, 858–872.

[gpr12883-bib-0057] Plenefisch T. and Bonjer K.P. 1997 The stress field in the Rhine Graben area inferred from earthquake focal mechanisms and estimation of frictional parameters. Tectonophysics 275, 7–97.

[gpr12883-bib-0058] Postma G.W. 1955 Wave propagation in a stratified medium. Geophysicists 20, 780–806.

[gpr12883-bib-0072] Rabbel W. 1994 Seismic anisotropy at the continental deep drilling site (Germany). Tectonophysics 232, 329–341, 10.1016/0040-1951(94)90094-9.

[gpr12883-bib-0059] Rabbel W. , Beilecke T. , Bohlen T. , Fischer D. , Frank A. , Hasenclever J. , *et al* 2004 Superdeep vertical seismic profiling at the KTB deep drill hole (Germany): seismic close‐up view of a major thrust zone down to 8.5 km depth. Journal of Geophysical Research 109, B09309.

[gpr12883-bib-0074] Röhr C. , Kohl J. , Hacker W. , Keyssner S. , Müller H. , Sigmund J. , Stroh A. and Zulauf G. 1990 German Continental Deep Drilling Program (KTB)—Geological survey of the pilot hole KTB Oberpfalz VB In: KTB REPORT 90‐8 (eds Emmermann R., Dietrich H.-G., Lauterjung J. and Wöhrl Th.), pp. B1–B55. Hannover.

[gpr12883-bib-0060] Rüger A. 2001 Reflection Coefficients and Azimuthal AVO Analysis in Anisotropic Media, Vol. 10 Tulsa, OK: Society of Exploration Geophysicists.

[gpr12883-bib-0061] Sambridge M. 1999 Geophysical inversion with a neighbourhood algorithm—I. Searching a parameter space, Geophysical Journal International 138, 479–494.

[gpr12883-bib-0062] Savage M.K. 1998 Lower crustal anisotropy or dipping boundaries? Effects on receiver functions and a case study in New Zealand. Journal of Geophysical Research 103, 15069–15087.

[gpr12883-bib-0063] Savage M.K. , Wessel A. , Teanby N.A. and Hurst A.W. 2010 Automatic measurement of shear wave splitting and applications to time varying anisotropy at Mt. Ruapehu volcano, New Zealand. Journal of Geophysical Research 115, B12321.

[gpr12883-bib-0064] Schulte‐Pelkum V. and Mahan K.H. 2014 A method for mapping crustal deformation and anisotropy with receiver functions and first results from US Array. Earth and Planetary Science Letters 402, 221–233.

[gpr12883-bib-0065] Schulte‐Pelkum V. , Monsalve G. , Sheehan A. , Pandey M.R. , Sapkota S. , Bilham R. , *et al* 2005 Imaging the Indian subcontinent beneath the Himalaya. Nature 435, 1222. 15988523 10.1038/nature03678

[gpr12883-bib-0066] Sherrington H.F. , Zandt G. and Frederiksen A. 2004 Crustal fabric in the Tibetan Plateau based on waveform inversions for seismic anisotropy parameters. Journal of Geophysical Research 109, B02312.

[gpr12883-bib-0067] Siegesmund S. , Vollbrecht A. , Chlupac T. , Nover G. , Dürrast H. , Müller J. , *et al* 1993 Fabric‐controlled anisotropy of petrophysical properties observed in KTB core samples. Scientific Drilling 4, 31–54.

[gpr12883-bib-0068] Stroh A. , Hansmann J. , Heinschild H.J. , Homann K.D. , Tapfer M. , Wittenbecker M. , *et al* 1990 Drill hole KTB Oberpfalz VB, Geoscientific investigations in the KTB‐field laboratory, depth interval 0–4000.1 m, Geochemistry/mineralogy. *KTB‐Report, 90–8*, C1–C37.

[gpr12883-bib-0069] Tsvankin I. , Gaiser J. , Grechka V. , van der Baan M. and Thomsen L. 2010 Seismic anisotropy in exploration and reservoir characterization: an overview. Geophysics 75, 75A15–75A29.

[gpr12883-bib-0070] Zang A. , Lienert M. , Zinke J. and Berckhemer H. 1996 Residual strain, wave speed and crack analysis of crystalline cores from the KTB‐VB well. Tectonophysics 263, 219–234.

[gpr12883-bib-0071] Zoback M.D. , Apel R. , Baumgärtner J. , Burdy M. , Emmermann R. , Engeser B. , *et al* 1993 Upper‐crustal strength inferred from stress measurements to 6 km depth in the KTB borehole. Nature 365, 633–635.

